# Removal of malachite green from aqueous solution using pulverized teak leaf litter: equilibrium, kinetic and thermodynamic studies

**DOI:** 10.1186/s13065-018-0448-8

**Published:** 2018-07-11

**Authors:** Emmanuel O. Oyelude, Johannes A. M. Awudza, Sylvester K. Twumasi

**Affiliations:** 1 Department of Chemistry, Kwame Nkrumah University of Science and Technology, Kumasi, Ghana; 2 Department of Applied Chemistry and Biochemistry, University for Development Studies, Navrongo Campus, P.O. Box 24, Navrongo, Ghana; 3 Faculty of Public Health, Catholic University College, Fiapre, Sunyani, Ghana

**Keywords:** Adsorption, Malachite green, Teak leaf litter, Isotherm, Kinetics, Thermodynamics

## Abstract

The removal of malachite green (MG) from aqueous solution using teak leaf litter powder (TLLP) was investigated. The process was influenced by initial concentration, pH and temperature of dye solution as well as TLLP dosage. Optimum removal of MG per gram of TLLP occurred at 2 g/L and at pH 6–8. Dubinin–Radushkevich and Freundlich isotherm models fit the batch adsorption data better than Langmuir isotherm. The monolayer capacity of TLLP was 333.33 mg/g at 293–313 K. The mean free energy of 7.07 kJ/mol implied physical adsorption. The pseudo-second order model fit the kinetic data better than the pseudo-first order model. Both intraparticle diffusion and film diffusion mechanisms jointly influenced the adsorption process but the latter was the rate-controlling step. Thermodynamic data indicated that the process was endothermic, spontaneous and feasible. Therefore, TLLP could be an important low-cost adsorbent for removal of MG from aqueous solution.

## Introduction

Malachite green (MG) is a synthetic triarymethane dye mainly employed for dyeing wool, silk, acrylic, leather, wood and paper [1]. It is also used in aquaculture as an ectoparasiticide and a fungicide because of its efficacy and low cost. The application of MG has been curtailed by some countries in recent years due to a number of toxicological concerns which are well documented. The dye is a possible carcinogen, tends to persist in the environment, and is toxic to aquatic and terrestrial organisms [2–4].

A number of methods are available for treating dye-impacted wastewater. However, adsorption method using activated carbon is popular due to its simplicity and efficacy [5]. The main impediment to unfettered employment of the method is the high cost of commercial activated carbon and the extra cost incurred in regenerating it. These have stimulated the interest of researchers to study non-conventional materials as cheaper and reliable substitutes for commercial activated carbon.

Forest plantations are established in Ghana mainly for production of fuel wood, electric poles, timber, environmental protection and reduction of rural poverty through employment generation. Teak, *Tectona grandis*, is among the most popular species of trees for reforestation in the country [6]. Plants contribute to nutrient cycling through litter fall. The factors that control litter production include: climate, age, size and species of trees; spacing of trees, type of forest, location and human activities [7, 8].

Rapid decomposition of litter assists to maintain soil fertility in tropical forest ecosystems [9]. The determinants of quality of any litter include: the specific weight and levels of carbon, nitrogen, lignin and polyphenols. Torreta and Takeda [10] indicated that, litter with C:N ratio greater than 30–40 may significantly reduce microbial activity leading to immobilization of nitrogen and impeded decomposition. Teak leaf litter (TLL) decomposes slowly due to a combination of its high C:N ratio, which is normally greater than 50; and high specific weight.

A comparison of the quantities of litter fall under monoculture teak plantation forests in Nigeria revealed that between 3774 and 6043 kg/ha litter was produced per annum [8]. Leaf litter accounts for at least 70% of the total litter fall [9]. It is estimated that an average of at least 3000 kg/ha of teak leaf litter is expected annually in Ghana. This important biomass is abundantly available and inexpensive [11] but currently either left unused or burnt. This research focused on the feasibility of employing pulverized TLL to remove MG dye from aqueous solution. The impacts of equilibrium adsorption, kinetic and thermodynamic parameters on the overall adsorption process were investigated to shed light on the nature of the adsorption process.

## Experimental

### Materials

TLL was collected from a monoculture teak plantation at Navrongo, north-eastern Ghana. The sample was washed continuously with large volume of tap water until the wash water was colorless and finally rinsed with distilled water. It was then air-dried for 10 days and crushed using a clean blender. The pulverized sample was washed repeatedly with distilled water until the wash water was colorless. The TLL sample was filtered, dried overnight in an oven at 105 °C. The cooled dry sample was then ground with a blender and sieved to obtain particles lesser than 210 µm. The sample was transferred into a glass bottle, tightly corked and labeled teak leaf litter powder (TLLP).

The MG (oxalate) dye used for the study was manufactured by Surechem Products Limited, Suffolk, England. The dye was used as supplied without any purification. A stock solution containing 1000 mg/L MG was prepared and dilute working solutions were prepared from the stock solution as appropriate. The maximum wavelength (λ_max_) of dilute MG solution was found to be 620 nm using UV/visible spectrophotometer (Jenway, model 6305). Concentrated hydrochloric acid and sodium hydroxide pellets used were manufactured by Panreac Quimica S.A., Barcelona, Spain. Distilled water was used for the preparation of all reagents.

### Adsorption equilibrium

Adsorption equilibrium tests were conducted for the removal of MG in aqueous solution using TLLP. Very dilute concentrations of the dye were first used to prepare a standard calibration plot use for the determination of the concentration of the dye samples. The effects of contact time, TLLP dose, pH of aqueous dye solution, temperature and concentration of MG dye were studied. For each test, a known mass of TLLP was weighed into a 250 mL stoppered Erlenmeyer flask, and a predetermined volume of MG solution of known concentration was added. The flask, with its content, was then shaken at 120 rpm and dye samples withdrawn at regular time intervals or after equilibrium as appropriate. The withdrawn sample was centrifuged at 5000 rpm for 5 min and the residual dye in the supernatant was determined by measuring its absorbance at 620 nm using UV/visible spectrophotometer (Jenway, model 6305). The quantity of MG, q_e_ (mg/g), removed from aqueous solution by TLLP was calculated from the following relationships:

1 $$q_{e} \, = \,\frac{{\left( {C_{0} \, - \,C_{e} } \right)V}}{w}$$

and

2 $$R\, = \,\frac{{\left( {C_{0} \, - \,C_{e} } \right)\, \times \,100}}{C_{0}}$$

where, C_0_ and C_e_ (mg/L) are the initial and equilibrium concentration of MG, respectively; V (L) is the volume of the dye, w (g) is the mass of TLLP used; q_e_ (mg/g) and R (%) is the quantity of MG removed from aqueous solution. All experiments were conducted at room temperature except for the study of the effect of temperature on the adsorption process. Each experiment was conducted in triplicate and the average values reported.

The effects of contact time and initial concentration of MG solution were studied together by adding 100 mL of dye solution to 1 g of TLLP in 250 mL Erlenmeyer flask. The initial concentration of the dye solution ranged between 50 and 200 mg/L. The impact of the dose of TLLP on removal of MG dye from aqueous solution was studied by fixing the initial concentration and volume of dye at 100 mg/L and 100 mL, respectively. The mass of TLLP was then varied from 0.05 to 1.00 g. The effect of pH of MG solution was examined by fixing the initial concentration of MG and mass of TLLP at 200 mg/L and 0.20 g, respectively. The pH of the dye solution was adjusted using 0.1 M HCl and 0.1 M NaOH solution. The initial volume of the dye used was 50 mL and the range of pH studied was 2–8 pH. The dye solution was partially decolorized and unstable at higher pH values. Calibrated pH meter (Crison, model Basic C20, Crison Instruments S.A., Barcelona, Spain) was used to take the readings. The effect of temperature of dye solution on its adsorption by TLLP was conducted by fixing initial concentration and volume of MG at 200 mg/L and 100 mL, respectively. The dye solution was initially fixed at pH 6 and the range of temperature studied was 20–40 °C.

### Adsorption kinetics

The adsorption kinetics experiments were conducted using initial MG concentrations of 200, 400 and 800 mg/L. The TLLP mass, temperature and initial pH of dye solution and volume of dye solution were kept constant at 1 g, 40 °C, 6.5 and 100 mL; respectively. The experiments were similar to those of batch equilibrium adsorption tests but dye samples were taken at regular intervals until the process reached equilibrium. The concentration of MG removed from aqueous solution by the adsorbent was determined using the equation below.

3 $$q_{t} \, = \,\frac{{\left( {C_{0} \, - \,C_{t} } \right)V}}{w}$$

where q_t_ (mg/g) is the quantity of MG solution at any time, C_o_ (mg/L) is the initial concentration of the aqueous solution of MG, C_t_ (mg/L) is the concentration of MG remaining in aqueous solution at any time, w is the mass of TLLP and V (L) is the volume of MG solution.

### Adsorption thermodynamics

The thermodynamics experiments were similar to the kinetic tests except that the temperature of dye solution was varied between 20 and 40 °C. The initial concentration and volume of the dye solution were fixed at 100 mg/L and 100 mL, respectively; the initial pH of dye solution was adjusted to 6.5 while the mass of TLLP used was fixed at 0.2 g. The concentration of the residual MG in solution was determined using Eq. (1).

## Results and discussion

### Effect of contact time and initial concentration of MG

**Fig. 1:**
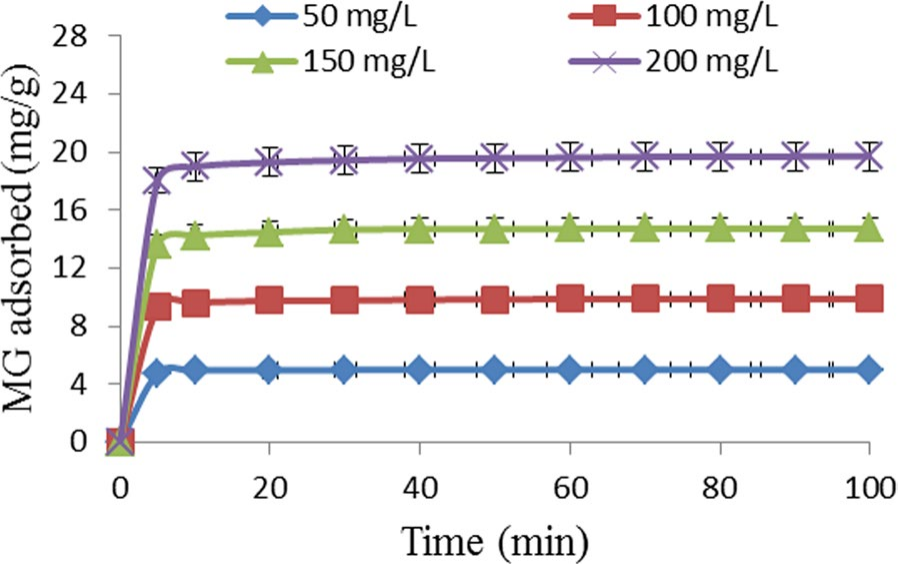
Effect of contact time and initial concentration of dye on removal of MG by TLLP

The plot of the effect of contact time and initial concentration of MG is presented in Fig. 1. The removal of MG from aqueous solution by TLLP was very rapid within the first 5 min before slowing down, and gradually became constant on attaining equilibrium. The rapid uptake of the dye during the first stage could be attributed to the availability of large number of sites on the surface of the adsorbent to facilitate the adsorption process. There was a marked reduction in the speed of adsorption during the second stage because of significant decrease in the number of vacant surface sites available for adsorption. There was equally repulsion between dye molecules already adsorbed on the surface of the adsorbent and dye molecules in the aqueous phase. Similar results have been reported by other researchers who studied the removal of MG from aqueous solution by adsorbents [1, 12].

The contact time required for the process to attain equilibrium was dependent on the initial concentration of MG in aqueous solution. For the initial MG concentration of 50, 100, 150, and 200 mg/L, the contact times required for the adsorption to attain equilibrium were: 30, 60, 70, and 90 min, respectively. The variation in the contact time required for adsorption to attain equilibrium could be explained on the basis of the boundary layer film the dye molecules must overcome to move from aqueous solution onto the surface of TLLP. Moreover, the dye molecules had to diffuse from the surface into the pores of the adsorbent. The more concentrated the dye solution, the more time it will take for dye molecules to move from the bulk solution into the pores of the adsorbent [13].

The adsorption capacity of TLLP was dependent on the initial concentration of the MG solution. The capacity of the adsorbent to remove dye molecules from solution increased from 4.99 to 19.70 mg/g when the initial concentration of MG solution was increased from 50 to 200 mg/L. These results could be interpreted in terms of concentration gradient. This provided the driving force to overcome resistances to mass transfer of dye molecules from the solution, toward the surface of the adsorbent [14, 15].

### Effect of TLLP dosage

**Fig. 2:**
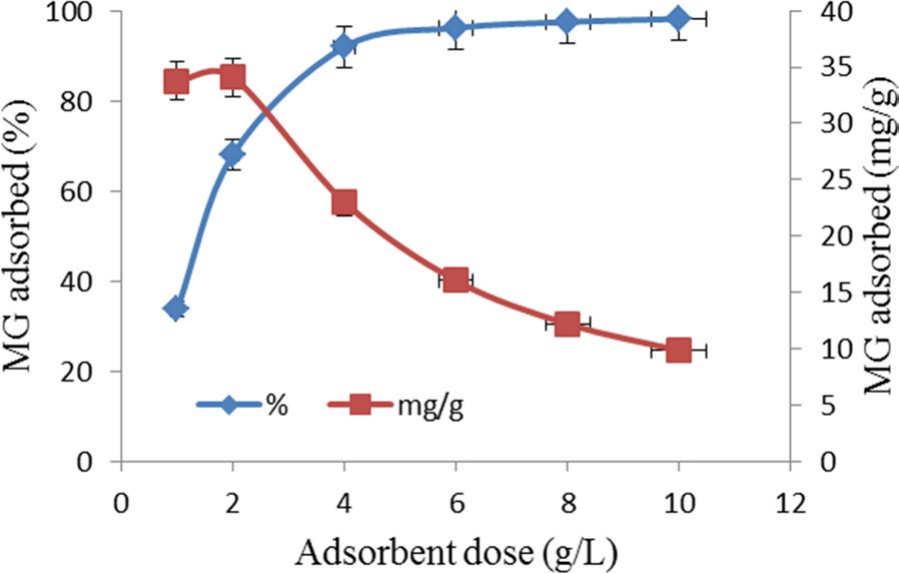
Effect of TLLP dosage on removal of MG from solution

The impact of TLLP dosage on the removal of MG from aqueous solution is shown in Fig. 2. The uptake of dye molecules increased from 33.76 to 98.19% as adsorbent dose was increased from 1 to 10 g/L. However, although the adsorption capacity increased marginally from 33.76 to 34.07 mg/g when the adsorbent dose was raised from 1 to 2 g/L, increase in dosage beyond 2 g/L led to continuous decrease in the adsorption capacity of the adsorbent. The observation could be attributed to rapid superficial adsorption onto the surface of the adsorbent as TLLP to MG concentration ratio increased. The superficial adsorption did not favor optimum uptake of the dye molecules by the adsorbent. This was responsible for the decrease in adsorption capacity of TLLP as dosage increased. Other researchers who observed similar phenomenon include Hamdaoui et al. [14], Sun et al. [15] and Oyelude et al. [16].

### Effect of pH of MG solution

**Fig. 3:**
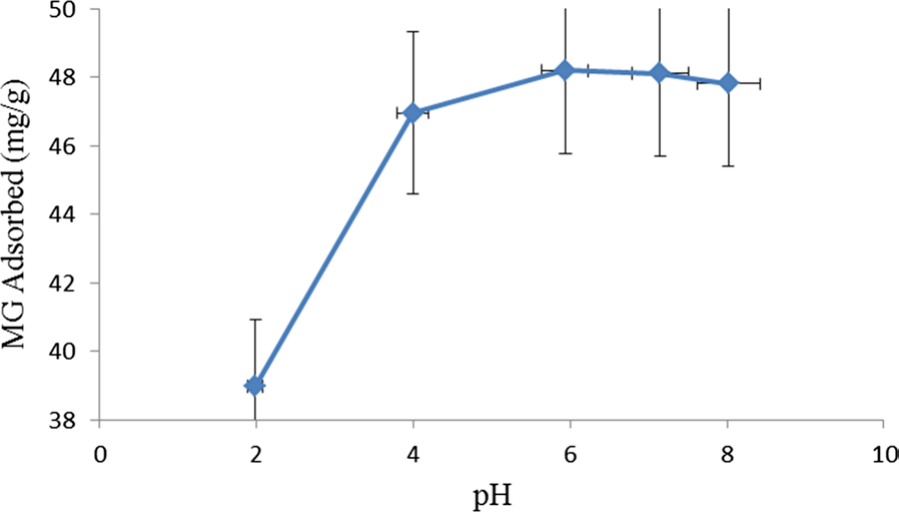
Effect of pH of dye solution on removal of MG

pH plays important role in adsorption. The effect of pH of MG solution on adsorption is presented in Fig. 3. The uptake of MG by TLLP decreased sharply below pH 6 but remained approximately constant from pH 6 to 8. The reduced uptake of the dye below pH 6 was due to electrostatic repulsion between positively charged surface of the adsorbent and the positively charged cationic MG dye. The number of positively charged sites on the adsorbent increased as the pH reduced. Hence the adsorption of the dye molecules to the surface of the adsorbent reduced as pH was lowered [1, 17, 18].

### Adsorption isotherms

An adsorbate may not interact with different adsorbents in the same way. Isotherms are plots used to express the distribution of adsorbate molecules between two phases with respect to time. The removal of MG from aqueous solution by TLLP was studied using isotherm models of Langmuir [19], Freundlich [20] and Dubinin–Radushkevich [21].

**Fig. 4:**
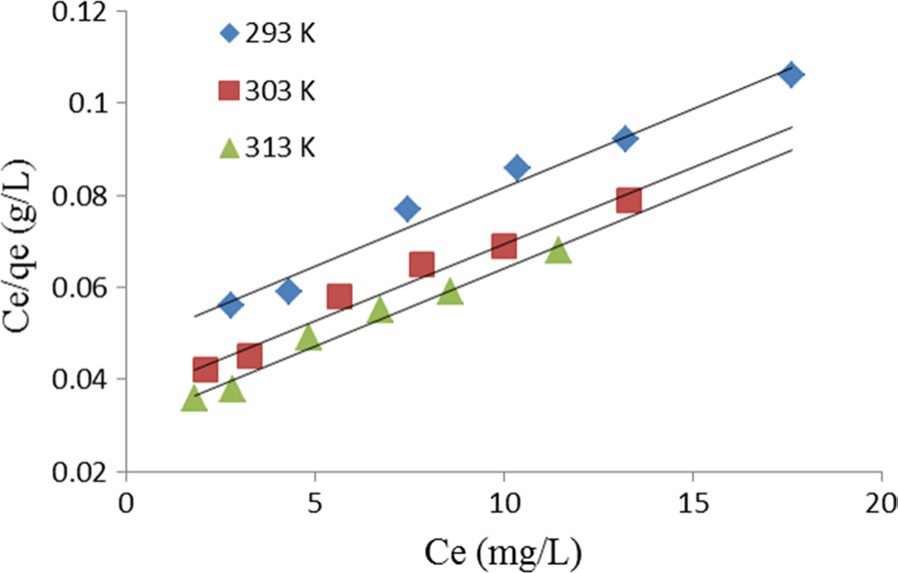
Linearized Langmuir isotherm plot for removal of MG by TLLP

Langmuir isotherm assumes constant adsorption energy and monolayer adsorption of adsorbate onto the surface of the adsorbent [19]. The linear form of the equation for the model is:

4 $$\frac{{C_{e} }}{{q_{e} }}\, = \,\frac{1}{{q_{m} }}C_{e} \, + \,\frac{1}{{q_{m} }}\frac{1}{{K_{L} }}$$

where C_e_ (mg/L) is the concentration of MG adsorbed at equilibrium, q_e_ (mg/g) is the mass of MG adsorbed at equilibrium per unit mass of TLLP, q_m_ (mg/g) is a constant related to the monolayer adsorption capacity of the adsorbent, and K_L_ (L/mg) is the Langmuir constant related to the rate of adsorption. A straight-line plot of C_e_/q_e_ versus C_e_ where slope equal to C_e_/q_e_ and intercept equals (1/q_m_)(1/K_L_) is presented in Fig. 4. The values of K_L_, q_m_, R_L_ and the linear correlation coefficient, R^2^, are presented in Table 1.

**Table 1:** Isotherm constants for the adsorption of MG onto TLLP at pH 6.5

Temperature (K)	K_L_ (L/mg)	q_m_ (mg/g)	R_L_	R^2^
Langmuir isotherm				
293	0.0638	333.33	0.0429	0.978
303	0.0833	333.33	0.0332	0.975
313	0.1000	333.33	0.0278	0.981

Temperature (K)	K_F_ (mg/g)	n	R^2^	
Freundlich isotherm				
293	26.1216	1.5267	0.994	
303	31.4051	1.5198	0.994	
313	35.2371	1.5314	0.993	

Temperature (K)	Β (mol^2^/J^2^)	$q_{DR}$ (mg/g)	E (kJ/mol)	R^2^
Dubinin–Radushkevich isotherm				
293	1.0 × 10^−8^	348.50	7.07	0.996
303	1.0 × 10^−8^	418.91	7.07	0.993
313	1.0 × 10^−8^	435.57	7.07	0.997

A dimensionless constant called separation factor, R_L_, can be used to explain the essential characteristics of Langmuir equation. R_L_ is defined as:

5 $$R_{L} \, = \,\frac{1}{{1\, + \,K_{L} C_{o} }}$$

where K_L_ is the Langmuir adsorption constant (L/mg) and C_o_ (mg/L) is the highest initial concentration of MG. The adsorption process is only favorable if 0 < R_L_ < 1, unfavorable if R_L_ > 1, linear if R_L_ = 1 and irreversible if R_L_ = 0. The value of RL for this present study was 0.0332 which indicates that the process was favorable.

**Fig. 5:**
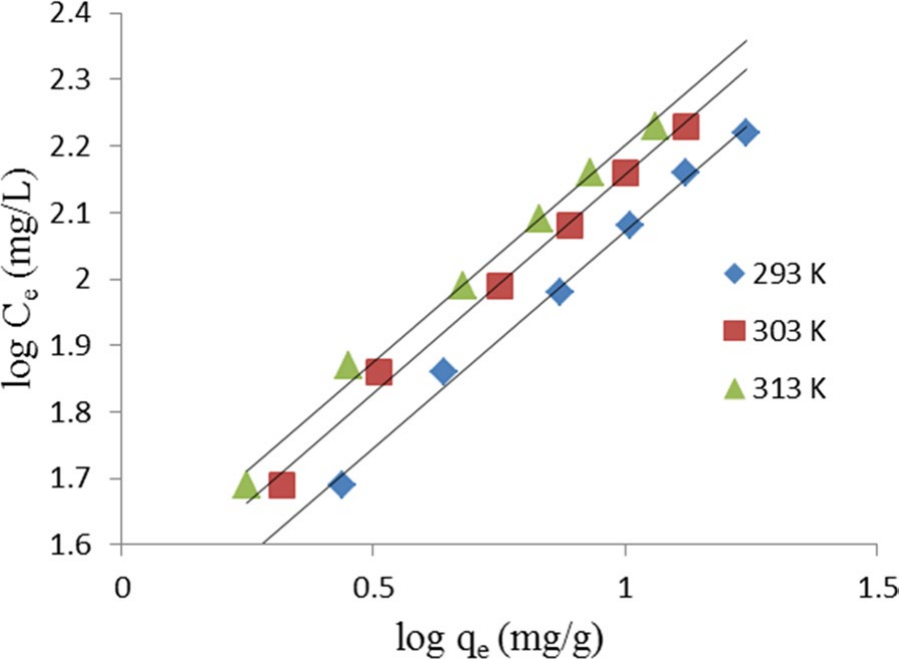
Linearized Freundlich isotherm plot for removal of MG by TLLP

Freundlich isotherm assumes adsorption from bulk solution onto an adsorbent with heterogeneous surface [20]. The linear form of the equation for the model is:

6 $$\log q_{e} \, = \,\frac{1}{n}logC_{e} \, + \,\log K_{F}$$

where q_e_ and C_e_ are as earlier defined, K_F_ (mg/g)(L/mg)^1/n^ is a constant representing the adsorbent capacity and 1/n is a constant the heterogeneity factor. The numerical value of 1/n must be lesser than one for the adsorption to be favorable. A linear plot of log q_e_ against log C_e_ is shown in Fig. 5.

The Dubinin–Radushkevich isotherm model [21] is used to determine the characteristic porosity of adsorbent and the mean free energy of adsorption. The isotherm assists to determine whether an adsorption is either physical or chemical in nature. The linear form of Dubinin–Radushkevich equation is:

7 $$\ln q_{e} = \ln q_{DR} - \beta \varepsilon^{2}$$

where $q_{DR}$ (mg/g) is the Dubinin–Radushkevich maximum monolayer adsorption capacity, β (mol^2^/J^2^) is a constant related to mean adsorption energy, and ε is the Polanyi potential which is calculated using the following relationship:

8 $$\varepsilon \, = \,RT\ln \left( {1\, + \,\frac{1}{{C_{e} }}} \right).$$

**Fig. 6:**
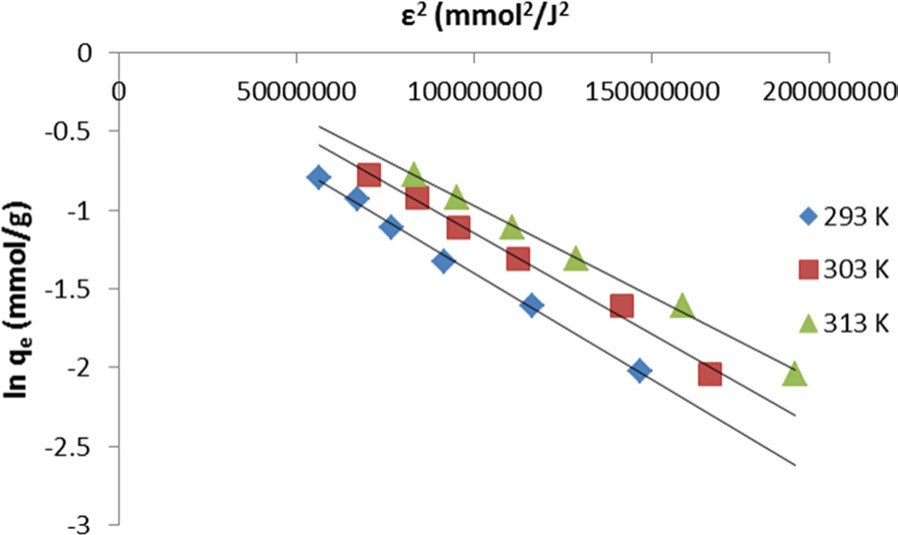
Linearized Dubinin–Radushkevich isotherm plot for removal of MG by TLLP

A plot of ln q_e_ against ε^2^ is presented in Fig. 6. The values of β and $q_{DR}$ were calculated from the slope and intercept of the plot, respectively. The mean free energy of adsorption is estimated from the value of β using the equation below.

9 $$E\, = \,\frac{1}{{\sqrt {2\beta } }}.$$

The value of E provides valuable information about the mechanisms of adsorption process. If E is lesser than 8 kJ/mol, the adsorption is regarded as physical in nature. However, if the value of E lies between 8 and 16 kJ/mol, the adsorption is regarded as chemical or ion exchange in nature [22]. The mean adsorption free energy, E, was calculated as 7.07 kJ/mol for this present study. This implies that the adsorption mechanism was physical in nature.

The summary of the isotherm constants and correlation coefficient, R^2^, for the three isotherm models applied for this study is presented in Table 1. On the basis of correlation coefficient alone, all the isotherm models fit the adsorption data well. However, Dubinin–Radushkevich isotherm fits best followed by Freundlich and Langmuir isotherms in that order.

The reported monolayer adsorption capacities of selected low-cost adsorbents for MG are presented in Table 2. TLLP is a good adsorbent for MG based on the basis of its adsorption capacity which was estimated to be 333.33 mg/g. It is worthy of note that temperature is one of the most important parameters that influence the uptake of dye molecules in aqueous solution. For this study, the uptake of MG from aqueous solution increased as temperature of dye solution increased irrespective of the initial concentration of the dye solution. This suggests that the adsorption process is endothermic in nature. This observation is attributed to the driving force of concentration gradient and increase in temperature which favored the endothermic process [23].

**Table 2:** Comparison of the reported maximum monolayer adsorption capacities of selected adsorbents for MG

Adsorbent	q_m_ (mg/g)	References
Teak leaf litter powder	333.33	This study
Commercial powder activated carbon	222.22	[24]
Dead leaves of plane tree	97.09	[14]
Chitosan beads	93.5	[25]
Bivalve shell-*Zea mays* L husk leaf	81.5	[18]
Rattan sawdust	62.71	[12]
Degreased coffee bean	55.3	[26]
Pineapple leaf powder	54.64	[27]

### Adsorption kinetics

**Fig. 7:**
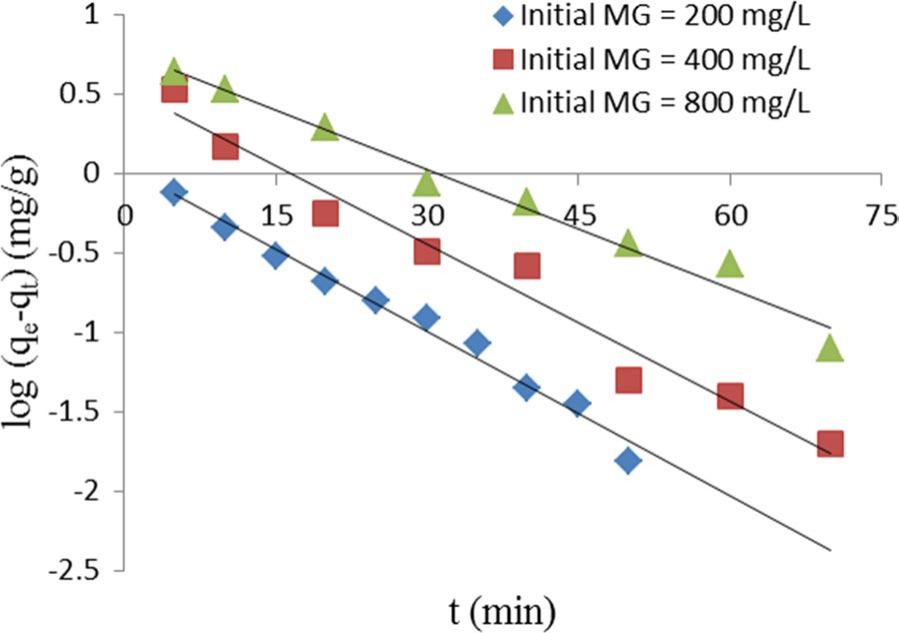
Pseudo-first order kinetic plot for removal of MG by TLLP

The kinetic of MG removal from aqueous solution were studied using pseudo-first order, pseudo-second order and intraparticle diffusion models. The equation for the pseudo-first order kinetic model [28] is:

10 $$\log \left( {q_{e} \, - \,q_{t} } \right)\, = \,\log q_{e} \, - \,\frac{{k_{1} t}}{2.303}$$

where q_e_ (mg/g) and q_t_ (mg/) are the quantity of dye adsorbed at equilibrium and time, t (min), respectively; and k_1_ (1/min) is the pseudo-first order rate constant. Figure 7 is a plot of log (q_e _− q_t_) against t. The values of k_1_ and q_e_ were determined from the slope (k_1_/2.303) and intercept (log q_e_), respectively. The R^2^ values obtained from the plot ranged from 0.970 to 0.983 which implies that the pseudo-first order kinetic model had good fit for the adsorption process. The values of k_1_, qe and R^2^ are shown in Table 3.

**Table 3:** Kinetic constants for removal of MG from aqueous solution by TLLP at different temperatures

Kinetic models	Co (mg/L)		
	200	400	800
Pseudo-first order			
q_e_, mg/g	1.102	3.524	5.929
k_1_, 1/min	0.015	0.014	0.010
R^2^	0.983	0.970	0.979
Pseudo-second order			
q_e_, mg/g	19.608	40.000	83.333
k_2_, g/mg min	0.260	0.089	0.029
h, mg/g min	99.963	142.880	199.999
R^2^	1.000	1.000	0.999
Intraparticle diffusion			
k_int_, mg/g min^1/2^	0.120	0.407	0.649
C, mg/g	18.730	36.190	73.570
R^2^	0.856	0.708	0.899

**Fig. 8:**
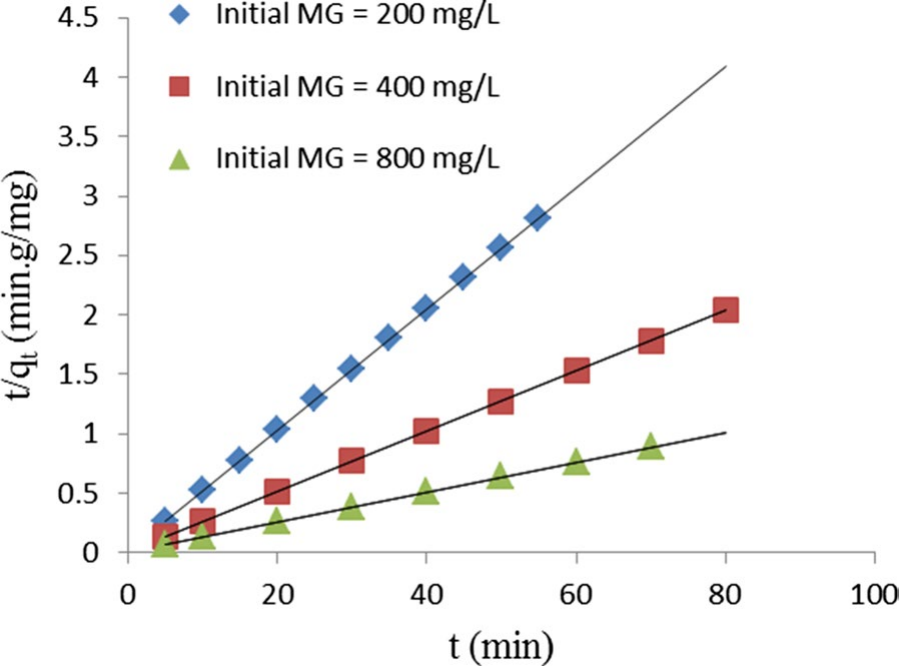
Pseudo-second order kinetic plot for removal of MG by TLLP

Ho and McKay [29] expressed the equation for the pseudo-second order kinetic as follows:

11 $$\frac{t}{{q_{e} }}\, = \,\frac{1}{{k_{2} q_{e}^{2} }}\, + \,\frac{1}{{q_{e} }}t$$

where k_2_ (g/mg min) is the rate constant. The plot of t/q_e_ against t is presented in Fig. 8 from which q_e_ and k_2_ are determined from the slope and the intercept, respectively. The initial rate of adsorption, h (mg/g min), is calculated from the following equation:

12 $$h\, = \,k_{2} q_{e}^{2} .$$

The values of R^2^ ranged between 0.999 and 1.000, which indicates that the adsorption of MG by TLLP perfectly fit the pseudo-second order kinetic model. The values of k_2_, q_e_, h and R^2^ are presented in Table 3.

**Fig. 9:**
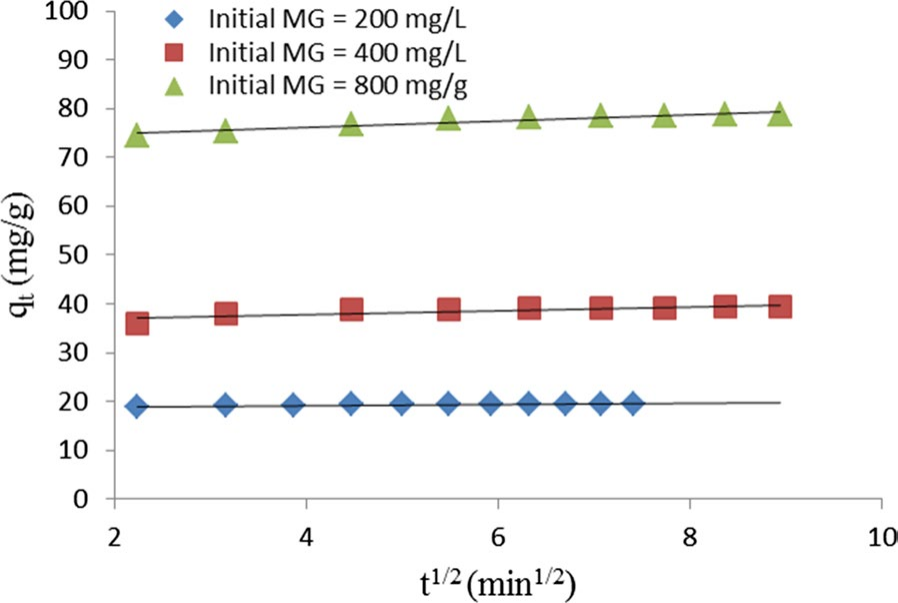
Intraparticle diffusion kinetic plot for removal of MG by TLLP

Intraparticle diffusion equation [30] is another important kinetic model commonly used to study adsorption kinetics. The intraparticle diffusion equation is:

13 $$q_{t} \, = \,k_{id} t^{1/2} \, + \,C$$

where k_id_ (mg/g min^1/2^) is the intraparticle diffusion rate constant, q_t_ (mg/g) is the quantity of dye adsorbed at time t (min), and C (mg/g) is the boundary layer thickness. The plot of q_t_ against t^1/2^ shown in Fig. 9 is linear for every initial concentration of MG implying that the adsorption process followed the intraparticle diffusion model. However, none of the plots passed through the origin indicating influence of boundary layer or film diffusion. The plot shows that the thickness of the boundary layer is proportional to the initial concentration of MG in aqueous solution. The values of k_id_, C and R^2^ determined from the plots are shown in Table 3.

### Adsorption mechanism

The mechanism for removal of dye molecules from aqueous solution may involve up to four steps. These steps include: bulk diffusion of molecules from solution to the surface of the adsorbent, boundary layer or film diffusion of molecules to the surface of the adsorbent, movement of molecules from the surface into the pores of the adsorbent or intraparticle diffusion and adsorption of dye molecules on active sites on the adsorbent through ion exchange, chelation and/or complexation [31].

It is clear from Fig. 9 that both intraparticle diffusion and film diffusion mechanisms take place at the same time in the uptake of MB by TLLP. The uptake of the dye by the adsorbent was very rapid within the first 5 min before slowing down, and gradually became constant on attaining equilibrium. Boyd model was used to further assess the kinetic data as to the rate-controlling step between intraparticle diffusion and film diffusion. The Boyd equation [32] is:

14 $${\text{F}}\, = \,1\, - \, \frac{6}{\pi }\mathop \sum \limits_{n\, = \,1}^{\infty } \frac{1}{{n^{2} }}{ \exp }\left( { - \,{\text{n}}^{2} {\text{Bt}}} \right)$$

and

15 $$F\, = \,\frac{{q_{t} }}{{q_{e} }}$$

where F equals the fractional attainment of equilibrium at time, t (min), n is the Freundlich constant, Bt is a function of F, and q_t_ (mg/g) and q_e_ (mg/g) represent quantity of dye adsorbed at time, t, and at equilibrium, respectively.

Reichenberg [33] proposed a simpler equation for calculating the values of Bt for each values of F > 0.85.

16 $$Bt\, = \, - \,0.4977\, - \,{ \ln }\left( {1\, - \,F} \right).$$

**Fig. 10:**
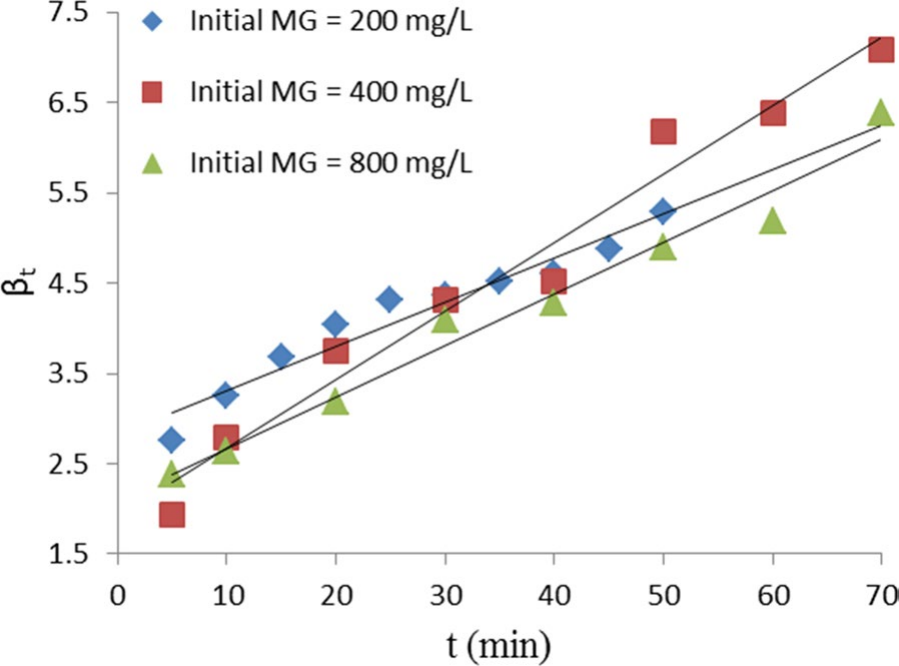
Boyd plot for removal of MG by TLLP

The plot of Bt versus t used to predict the mechanism of the adsorption process is presented in Fig. 10. The linear plot did not pass through the origin for every initial concentration of the dye in aqueous solution. This confirms that film diffusion was the rate-controlling step in the uptake of MG in aqueous solution by TLLP.

### Adsorption thermodynamics

Standard enthalpy (∆H°, kJ/mol), standard entropy (∆S°, J/mol K), and standard free energy (∆G°, kJ/mol), are vital thermodynamics parameters that must be considered for proper assessment of any adsorption process. The following equations were employed to estimate their values for the studied temperature ranging between 293 and 313 K.

17 $$\Delta G^\circ \, = \, \Delta H^\circ \, - \,T\Delta S^\circ$$

18 $$\Delta G^\circ \, = \, - \,RTlnK_{d}$$

where R is the gas constant (8.314 J/mol K), T (K) is temperature and

19 $$K_{d} \, = \,{\raise0.7ex\hbox{${q_{e} }$} \!\mathord{\left/ {\vphantom {{q_{e} } {C_{e} }}}\right.\kern-0pt} \!\lower0.7ex\hbox{${C_{e} }$}}$$

where K_d_ is the distribution coefficient, q_e_ (mg/g) is the quantity of MG adsorbed at equilibrium and C_e_ (mg/L) is the quantity of MG remaining in solution at equilibrium. Equation (18) was used to estimate the values of ∆G° at various temperatures. The equalization of Eqs. (17) and (18) produce:

20 $$\ln K_{d} \, = \,\frac{\Delta S^\circ }{R}\, - \,\frac{\Delta H^\circ }{RT}.$$

**Fig. 11:**
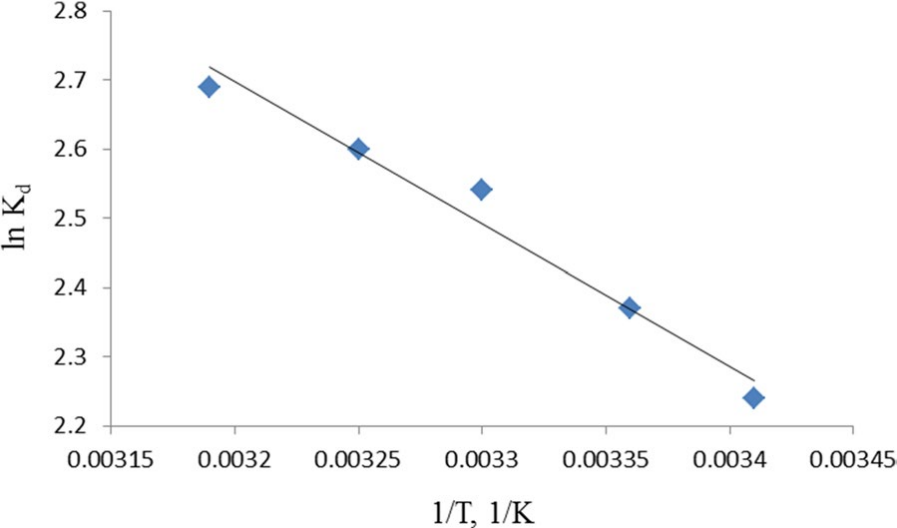
Plot of ln Kd versus 1/T for removal of MG by TLLP

The plot of ln K_d_ versus 1/T shown in Fig. 11 is used for the estimation of the magnitudes of ∆H° and ∆S°. The values of ∆H°, ∆S° and ∆G° are presented in Table 4. The values of ∆G° were negative at the range of temperature studied implying that the adsorption process was spontaneous and thermodynamically favorable. However, the positive value of ∆H° was positive indicating an endothermic process. The positive value of ∆S° was a reflection of the increased randomness at the TLLP/MG solution interface due to the affinity of the adsorbent for the dye.

**Table 4:** Thermodynamic parameters for the removal of MG from aqueous solution by TLLP

∆H° (kJ/mol)	∆S° (kJ/mol K)	∆G° (kJ/mol)				
		293 K	298 K	303 K	308 K	313 K
17.069	0.077	− 1.964	− 2.138	− 2.348	− 2.447	− 2.575

## Conclusion

The removal of MG from aqueous solution revealed that the process was influenced by initial concentration, pH and temperature of dye solution as well as TLLP dosage. Optimum uptake of the dye per gram of the adsorbent occurred at 2 g/L and at pH 6–10. Dubinin–Radushkevich and Freundlich isotherm models fit the batch adsorption data better than Langmuir isotherm. However, the monolayer capacity of TLLP for the removal of MG in aqueous solution was calculated to be 333.33 mg/g at 293–313 K. The adsorption process was physical in nature because the mean free energy was 7.07 kJ/mol.

The pseudo-second order model fit the kinetic data much better than the pseudo-first order model. Intraparticle diffusion and film diffusion jointly influence the mechanism of adsorption. However, film diffusion was the rate-controlling step for the uptake of MG in aqueous solution by TLLP. Thermodynamic data indicated that the process was endothermic, spontaneous and feasible. Therefore, TLLP could be an important low-cost adsorbent for removal of MG from aqueous solution.

## Abbreviations

MG: malachite green
TLL: teak leaf litter
TLLP: teak leaf litter powder
C:N: carbon to nitrogen ratio
q_e_: mass of MG (mg) per gram of TLLP at equilibrium
q_t_: mass of MG (mg) per gram of TLLP at any time
C_o_: initial concentration of MG (mg/L)
C_e_: concentration of MG remaining in aqueous solution at equilibrium (mg/L)
C_t_: concentration of MG remaining in aqueous solution at any time (mg/L)
t: time (min)
V: volume of aqueous solution of MG (L)
R: proportion of MG removed from aqueous solution (%)
T: temperature (Kelvin)
q_m_: Langmuir isotherm monolayer adsorption capacity of TLLP (mg/g)
K_L_: Langmuir isotherm constant
R_L_: linear correlation coefficient (R^2^)
K_F_: Freundlich isotherm constant
1/n: heterogeneity factor of Freundlich isotherm
q_DR_: Dubinin–Radushkevich monolayer adsorption capacity of TLLP (mg/g)
β: constant related to mean free energy of adsorption
ε: Polanyi potential
R: gas constant (8.314 J/mol K)
E: mean free energy of adsorption
k_1_: pseudo-first order kinetic rate constant
k_2_: pseudo-second order kinetic rate constant
h: initial rate of adsorption (mg/g min)
k_id_: intraparticle diffusion rate constant
F: fractional attainment of equilibrium
Bt: function of F
ΔH°: change in standard enthalpy
ΔS°: change in standard entropy
ΔG°: change in standard free energy
K_d_: distribution coefficient

## References

[1] Ahmad MA, Alrozi R (2011). Removal of malachite green dye from aqueous solution using rambutan peel-based activated carbon: equilibrium, kinetic and thermodynamic studies. Che Eng J.

[2] Alderman DJ (1985). Malachite green: a review. J Fish Dis.

[3] Culp SJ, Beland FA (1996). Malachite green: a toxicological review. J Am Col Toxicol.

[4] Srivastava S, Sinha R, Roy D (2004). Toxicological effects of malachite green. Aquatic Toxicol.

[5] Tan IAW, Ahmad AL, Hameed BH (2008). Adsorption of basic dye on high-surface-area activated carbon prepared from coconut husk: equilibrium, kinetic and thermodynamic studies. J Hazard Mat.

[6] Forestry Commission (2016) Ghana Forest Plantation Strategy: 2016–2040. http://www.fcghana.org/userfiles/files/Plantation%20Annual%20Report/Ghana%20Forest%20Plantation%20Strategy_24_01_16(2).pdf. Accessed 3 Feb 2017

[7] O’Connell AM, Sankaran KV, Nambiar EKS, Brown AG (1997). Organic accretion, decomposition and mineralization. Management of soil nutrient and water in tropical plantation forest.

[8] Ojo AF, Kadeba TOS, Kayode J (2010). Litter mass and nutrient dynamics in a transformed rain forest ecosystem in southwestern Nigeria. Bangladesh J Sci Ind Res.

[9] Thakur TK, Thakur A (2014). Litter fall patterns of a dry tropical forest ecosystem of central India. Ecol Environ Conserv.

[10] Torreta NK, Takeda H (1999). Carbon and nitrogen dynamics of decomposing leaf litter during a 3.5-year decomposition. Eur J Soil Bio.

[11] Bello OS, Ahmad AM (2012). Coconut (*Cocos nucifera*) shell based activated carbon for the removal of malachite green from aqueous solution. Sep Sci Technol.

[12] Hameed BH, El-Khaiary MI (2008). Equilibrium, kinetics and mechanism of malachite green adsorption by activated carbon prepared from bamboo by K_2_CO_3_ activation and subsequent gasification with CO_2_. Hazard Mat.

[13] Hameed BH, Salman JM, Ahmad AL (2009). Adsorption isotherm and kinetic modeling of 2,4-d pesticide on activated carbon derived from date stones. J Hazard Mat.

[14] Hamdaoui O, Saoudi F, Chiha M, Naffrechoux E (2008). Sorption of malachite green by a novel sorbent, dead leaves of plane tree: equilibrium and kinetic modeling. Che Eng J.

[15] Sun X-F, Wang S-G, Liu X-W, Gong W-X, Bao N, Gao B-Y, Zhang H-Y (2008). Biosorption of malachite green from aqueous solutions onto aerobic granules: kinetic and equilibrium studies. Bioresour Technol.

[16] Oyelude EO, Frimpong F, Dawson D (2015). Studies on the removal of basic fuchsin dye from aqueous solution by HCl treated malted sorghum mash. J Mater Environ Sci.

[17] Akar E, Altinişik A, Seki Y (2013). Using activated carbon produced from spent tea leaves for the removal of malachite green from aqueous solution. Ecol Eng.

[18] Jalil AA, Triwahyono S, Yaakob MR, Azmi ZZA, Sapawe N, Kamarudin NHN, Setiabudi HD, Jaafar NF, Sidik SM, Adam SH, Hameed BH (2012). Utilization of bivalve shell-treated *Zea mays L*. (maize) husk leaf as a low-cost biosorbent for enhanced adsorption of malachite green. Bioresour Technol.

[19] Langmuir L (1918). The adsorption of gases on plane surfaces of glass, mica and platinum. J Am Chem Soc.

[20] Freundlich HMF (1906). Over the adsorption in solution. Journal of Phy Chem.

[21] Dubinin MM, Radushkevich L (1947). The equation of the characteristic curve of activated charcoal. Proceed Acad Sci Physical Chem Sec.

[22] Javadian H, Ghorbani F, Tayebi H-A, Asl SMH (2015). Study of the adsorption of Cd(II) from aqueous solution using zeolite-based geopolymer, synthesized from coal fly ash; kinetic, isotherm and thermodynamic studies. Arab J Chem.

[23] Bello OS, Ahmad MA, Ahmad M (2012). Adsorptive features of banana (*Musa paradisiaca*) stalk-based activated carbon for malachite green dye removal. Chem Ecol.

[24] Malik R, Ramteke DS, Wate SR (2007). Adsorption of malachite green on groundnut shell waste based powdered activated carbon. Waste Mgt.

[25] Bekçi Z, Ӧzveri C, Seki Y, Yurdakoç K (2008). Sorption of malachite green on chitosan bead. J Hazard Mat.

[26] Baek M-H, Ijagbemi CO, Se-Jin O, Kim D-S (2010). Removal of malachite green from aqueous solution using degreased coffee bean. J Hazard Mat.

[27] Chowdhury S, Chakraborty S, Saha P (2011). Biosorption of Basic Green 4 from aqueous solution by Ananas comosus (pineapple) leaf powder. Colloids Surf B Biointerfaces.

[28] Lagergren S (1898). About the theory of so-called adsorption of soluble substances. Kungliga SvenskaVetenskapsakademiens Handlingar.

[29] Ho YS, McKay G (1999). Pseudo-second order model for sorption processes. Process Biochem.

[30] Weber WJ, Morris JC (1963). Intraparticle diffusion during the sorption of surfactants onto activated carbon. J Sanit Eng Div Am Soc Civ Eng.

[31] Cheung WH, Szeto YS, MacKay G (2007). Intraparticle diffusion processes during acid dye adsorption onto chitosan. Bioresour Technol.

[32] Boyd GE, Adamson AW, Myers LS (1947). The exchange adsorption of ions from aqueous solutions by organic zeolites. Part II: kinetics. J Am Chem Soc.

[33] Reichenberg D (1953). Properties of ion exchange resins in relation to their structure. Part III: kinetics of exchange. J Am Chem Soc.

